# Cox-Maze III procedure with valvular surgery in an autopneumonectomized patient

**DOI:** 10.1186/1749-8090-7-116

**Published:** 2012-11-08

**Authors:** Jin Hong Wi, Ho-Ki Min, Do Kyun Kang, Hee Jae Jun, Youn-Ho Hwang, Dong-Kie Kim, Hyun Kuk Kim, Hang Jea Jang, Il Rhee

**Affiliations:** 1 Central Physical Examination Center of Military Manpower Administration, Seoul, South Korea; 2 Department of Thoracic and Cardiovascular Surgery, Haeundae Paik Hospital, Inje University College of Medicine, 875 (Jwadong) Haeundae-ro, Haeundaegu, Busan, 612-030, South Korea; 3 Department of Internal Medicine, Haeundae Paik Hospital, Inje University College of Medicine, Busan, South Korea; 4 Department of Internal Medicine, Dong-Eui Medical Center, Busan, South Korea

**Keywords:** Arrhythmia therapy, Mitral valve repair, Pneumonectomy

## Abstract

Destructive pulmonary inflammation can leave patients with only a single functional lung, resulting in anatomical and physiological changes that may interfere with subsequent cardiac surgeries. Such patients are vulnerable to perioperative cardiopulmonary complications. Herein, we report the first case, to our knowledge, of an autopneumonectomized patient who successfully underwent a modified Cox-Maze III procedure combined with valvular repairs. The three major findings in this case can be summarized as follows: (1) a median sternotomy with peripheral cannulations, such as femoral cannulations, can provide an optimal exposure and prevent the obstruction of vision that may occur as a result of multiple cannulations through a median sternotomy; (2) a modified septal incision combined with biatrial incisions facilitate adequate exposure of the mitral valve; and (3) the aggressive use of intraoperative ultrafiltration may be helpful for the perioperative managements as decreasing pulmonary water contents, thereby avoiding the pulmonary edema associated with secretion of inflammatory cytokines during a cardiopulmonary bypass. We also provide several suggestions for achieving similar satisfactory surgical outcomes in patients with a comparable condition.

## Background

Severe destructive lung lesions may form as sequelae of inflammatory pulmonary disease. An extreme sequela is autopneumonectomy, which results in anatomical and physiological changes that can have deleterious effects on subsequent cardiac surgery, especially when multiple procedures are required. Herein, we report our success performing a modified Cox-Maze III procedure combined with valvular surgery in an autopneumonectomized patient.

## Case presentation

**Figure 1:**
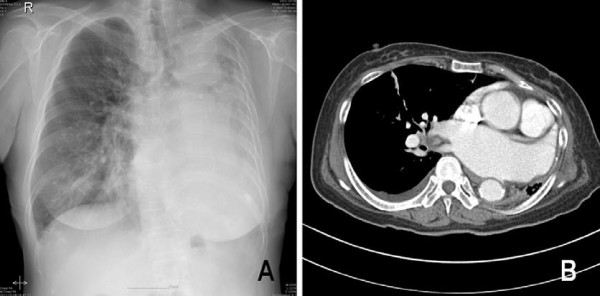
**Images of the patient’s chest prior to surgery.** (**A**) Preoperative chest X-ray and (**B**) enhanced computed tomographic scan with axial image.

**Figure 2:**
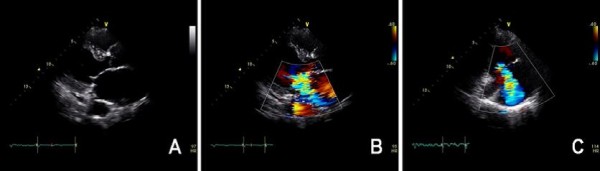
**Preoperative transthoracic echocardiographic images.** (**A**) The parasternal long-axis view shows the prolapsed anterior leaflet of the mitral valve, while (**B**) color flow mapping reveals severe regurgitation of the mitral valve. (**C**) The modified two-chamber view with color flow mapping reveals moderate to severe regurgitation of the tricuspid valve.

The patient was a 66-year-old woman with a 40-year history of pulmonary tuberculosis; she was referred after experiencing progressive dyspnea and easy fatigability for 2 months. Three years earlier, she had been diagnosed with valvular heart disease and atrial fibrillation. Prior to being referred, she had progressed to New York Heart Association (NYHA) Class IV heart failure despite maximal medical management. Analysis of the patient’s blood gases showed normal pH (7.40), mild hypercapnea (PaCO_2_ = 45 mmHg), and hypoxemia (PaO_2_ = 74 mmHg). Chest X-ray and computed tomography (CT) revealed a destroyed and totally shrunken left lung, a hyperinflated right lung crossing the midline, and mediastinal structures severely displaced to the left hemithorax (Figure 1). Pulmonary function tests revealed a severe obstructive pattern with restrictive defect: Forced vital capacity (FVC) was 1.29 L (43% of the predicted value), forced expiratory volume in 1 second (FEV1) was 0.80 L (35% of the predicted value), and FEV1/FVC was 62%. An electrocardiogram showed atrial fibrillation with rapid ventricular response (103 beats per minute). An echocardiogram revealed severe mitral regurgitation and a dilated left atrium (52 mm), moderate to severe tricuspid regurgitation, increased right ventricular systolic pressure (38 mmHg), and preserved left ventricular function (Figure 2). Right heart catheterization revealed that pulmonary artery pressure was 42/21 mmHg, with mean pulmonary artery pressure of 28 mmHg. We decided that surgery was required, since the patient had been suffering from progressive symptoms and had engaged in an active lifestyle prior to the onset of symptoms. Before the operation, the patient began vigorous chest physiotherapy and underwent forced diuresis.

**Figure 3:**
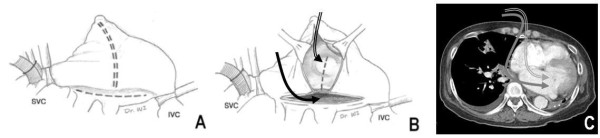
**Modified incisions used during the patient’s surgery.** (**B**) An additional septal incision can facilitate exposure of the mitral valve. (**B**, **C**) A left atrial incision (solid arrow) would not provide as much visibility of the mitral valve as the modified septal incision (double arrow). Thus, the latter was used to maximize the surgeon’s view during the procedure. These incisions also were used for the modified Cox-Maze III operation. IVC: inferior vena cava; SVC: superior vena cava.

During the surgery, we cannulated the right femoral artery and vein. Median sternotomy revealed that the right lung was crossing the midline, while the heart was markedly displaced and rotated into the left hemithorax. Supplementalrily we directly cannulated the superior vena cava and performed a cardiopulmonary bypass (CPB). During the CPB, we used aggressive intraoperative ultrafiltration to both reduce lung water content and pulmonary vascular resistance. After antegrade cardioplegia delivery without topical cooling, the right atrium was incised from the tip of the appendage to the mid-portion of the interatrial groove. Additionally, a left atrial incision was made parallel to the interatrial groove and connected to the right atrial incision. The additional incision was made across the interatrial septum to the fossa ovalis to grant the surgeon better visibility of the mitral valve and to facilitate the valve’s exposure (Figure 3). We inspected the mitral annular dilatation and the prolapsed A2 portion with degenerative change; this examination revealed that the tricuspid annulus was markedly dilated. We then performed a modified Cox-Maze III procedure using cryoablation. Thereafter, 5/0 Prolene sutures were employed to perform an Alfieri stitch repair of the mitral cusp. The mitral annulus was stabilized with a Cosgrove-Edwards ring, and tricuspid annuloplasty was performed with an Edwards MC3 ring. CPB was discontinued without any difficulty. Total bypass time was 209 min and aortic cross clamp time was 173 min.

The patient was extubated 14 hours after the operation. The postoperative course was unremarkable and she was discharged on the 11th postoperative day. At the 11-month follow-up, she had normal sinus rhythm, no mitral regurgitation (pressure gradient: 3.9 mmHg), and mild tricuspid regurgitation; she is currently classified as having NYHA class II heart failure. At the last follow-up, she was prescribed an angiotensin II receptor blocker, diuretics, and bronchodilator medications.

### Discussion

Patients with pulmonary dysfunction are known to be vulnerable to perioperative cardiopulmonary complications. Lethal outcomes may result from even minor atelectasis, congestion, or nasocomial infection. Thus, it can be challenging to treat these patients, and it is important to consider selection criteria, exposure of intracardiac structures, avoidance of the phrenic nerve and remaining lung injuries, and perioperative management. Unfortunately, literature on cardiac surgeries in autopneumonectomized patients is lacking [1,2]; in order to develop our surgical plan, we had to rely almost completely on reports of cardiac surgeries performed after pneumonectomy.

Our literature review identified several potential methods of determining whether the patient possessed the minimal pulmonary reserve required to avoid dependency on mechanical ventilation after operation; these included a pulmonary function test, an arterial blood gas analysis, and pulmonary artery pressure [3,4]. Previous authors have recommended that patients not undergo operation if their forced expiratory volume per 1 second is <40% of predicted values and <800 ml, if their resting carbon dioxide tension is >50 mmHg, or if their diffusion capacity of carbon monoxide is >50% of predicted values. Further, surgery is known to be riskier in patients with a mean pulmonary artery pressure >40 mmHg. Judging by these criteria, our patient was only marginally acceptable. However, we decided to proceed with surgery since she had been suffering from acutely progressive symptoms and had maintained an active lifestyle before the onset of symptoms.

An autopneumonectomized lung can displace and rotate the heart into the ipsilateral hemithorax, thereby making exposure cumbersome through the conventional approach. As a result, it may be preferable to use an alternative surgical approach after pneumonectomy [5]. However, it is also feasible to perform median sternotomy, which is a more familiar way to conduct cardiac surgery after pneumonectomy [1,2,6]; we decided to perform a median sternotomy with femoral cannulations. We hoped that the use of a median sternotomy with peripheral cannulations would prevent the surgical field from becoming crowded with cannulae; additionally, we anticipated it would improve exposure, since a large number of cannulae and surgical instruments in the operative field would limit a surgeon’s vision from the right side when the heart is severely rotated into the left side. A modified septal incision combined with biatrial incisions also seemed likely to facilitate exposure of the mitral valve, since the left atrial structures were located farther far away from the surgeon’s vision than those on the right (Figure 3).

The Cox-Maze III procedure, which may be modified in a number of ways, is a well-established technique for restoring sinus rhythm in patients with atrial fibrillation combined with mitral valve disease [7]. Cardioversion to sinus rhythm improves hemodynamics and ventilation efficiency [8]. In our opinion, it is important to restore sinus rhythm when pulmonary reserves are diminished, as in the current case. The biatrial Cox-Maze III procedure was successful for our patient, whose heart rhythm has been maintained since the surgery.

This procedure has been associated with injuries to the phrenic nerve and the remaining lung. Diaphragmatic dysfunction can be minimized by avoidance of topical cardiac hypothermia [1,2,6]. It is also advisable to avoid use of a Swan-Ganz catheter, and to place the central line on the contralateral side of the normal hemithorax [3,6]. Additionally, we recommend the aggressive use of intraoperative ultrafiltration (IU), a technique that has been known to remove mediators of inflammation (i.e., cytokines), as well as accumulations of tissue water generated during CPB. IU can be used not only to decrease pulmonary vascular resistance and lung water content, but also to conserve blood and improve PaO_2_[9]. We also advocate intensive chest physiotherapy, low-pressure ventilation, and early extubation during the perioperative period. Fluid administration should be restricted and adjusted perioperatively depending on volume status. Early postoperative mobilization may also be helpful [1-3].

## Conclusions

To the best of our knowledge, this report is the first to describe the use of a modified Cox-Maze III procedure combined with valvular repairs in a case of autopneumonectomized lung. In patients with this condition, all possible measures should be employed to preserve the single functional lung. We especially recommend not only an individualized approach, but also the aggressive use of intraoperative ultrafiltration to reduce lung water content and restore remaining pulmonary function. Our results suggest that, with optimal perioperative and intraoperative management, it is possible to achieve an acceptable outcome following cardiac surgery in autopneumonectomized patients; prior to the procedure, however, patients should be evaluated with appropriate selection criteria and provided with adequate counseling.

## Consent

Written informed consent was obtained from the patient for publication of this case report and all accompanying images. A copy of the written consent is available for review by the Editor-in-Chief of this journal.

## Competing interests

The authors declare that they have no competing interests.

## Author information

HM - Is a surgeon of the Thoracic and Cardiovascular Surgery department at the Haeundae Paik Hospital where the patient underwent operation. He is also an assistant professor at the Inje University College of Medicine in Busan, Korea.

## Authors’ contributions

JW: participated in the management and wrote the manuscript. HM: performed surgery on the patient and revised the manuscript. DK: revised the manuscript. HJ: revised the manuscript. YH: revised the manuscript. DK: revised the manuscript. HK: revised the manuscript. HJ: revised the manuscript. IR: revised the manuscript. All authors read and approved the final manuscript.
